# Production of copper-64 using a hospital cyclotron: targetry, purification and quality analysis

**DOI:** 10.1097/MNM.0000000000001422

**Published:** 2021-05-05

**Authors:** Maite Jauregui-Osoro, Simona De Robertis, Philip Halsted, Sarah-May Gould, Zilin Yu, Rowena L Paul, Paul K Marsden, Antony D Gee, Andrew Fenwick, Philip J. Blower

**Affiliations:** a School of Biomedical Engineering and Imaging Sciences, King’s College London, School of Biomedical Engineering and Imaging Sciences, St Thomas’ Hospital; b National Physical Laboratory, Teddington, Middlesex, London, UK

**Keywords:** copper-64, purification, quality control, solid targetry

## Abstract

To construct and evaluate a ^64^Cu production system that minimises the amount of costly ^64^Ni, radionuclidic impurities and nonradioactive metal contamination and maximises radiochemical and radionuclidic purity and molar activity; and to report analytical and quality control methods that can be used within typical PET radiochemistry production facilities to measure metal ion concentrations and radiometal molar activities.

**Methods:**

Low volume was ensured by dissolving the irradiated nickel in a low volume of hydrochloric acid (<1 mL) using the concave gold target backing as a reaction vessel in a custom-built target holder. Removal of contaminating ^55^Co and nonradioactive trace metals was ensured by adding an intermediate hydrochloric acid concentration step during the conventional ion-exchange elution process. The radionuclidic purity of the product was determined by half-life measurements, gamma spectroscopy and ion radiochromatography. Trace metal contamination and molar activity were determined by ion chromatography.

**Results and conclusions:**

On a small scale, suitable for preclinical research, the process produced typically 3.2 GBq ^64^Cu in 2 mL solution from 9.4 ± 2.1 mg nickel-64 electroplated onto a gold target backing. The product had high molar activity (121.5 GBq/µmol), was free of trace metal contamination detectable by ion chromatography and has been used for many preclinical and clinical PET imaging applications.

## Abstract 2

Supplemental Digital Content is available in the text.

## Introduction

Copper-64 (^64^Cu) is an intermediate half-life positron-emitting radionuclide (t_1/2_ = 12.7 hours), suitable for the radiolabelling of biomolecules with slow targeting kinetics (e.g. peptides/proteins, antibodies) for both diagnostic and therapeutic purposes. Its short positron range (similar to that of ^18^F) [1–3] allows for high-resolution PET imaging. ^64^Cu has a low positron yield (18%), as it also decays by beta emission (39%) and electron capture accompanied by Auger electron emission (43%); these decay modes potentially allow ^64^Cu to be used for targeted radionuclide therapy. ^64^Cu radiopharmaceuticals can thus be used for quantitative PET imaging to calculate radiation dosimetry prior to performing targeted radiotherapy with ^64^Cu or its beta-emitting isotopologue ^67^Cu. ^64^Cu has been incorporated into many labelled bioconjugates based on antibodies, peptides and small molecules that target specific receptors or antigens, particularly in oncology applications. The most successful bifunctional chelators used in these bioconjugates are macrocyclic polyamine and polyaminocarboxylate ligands [4–7]. ^64^Cu, in the form of bis(thiosemicarbazone) complexes, has also been used for PET imaging of hypoxia [8–10], blood flow [11] changes in copper trafficking [12–15] and cell tracking [16]. Free unchelated copper (e.g. ^64^CuCl_2_) has also been used to measure copper trafficking in vivo to understand better the physiology of copper metabolism in health and disease, particularly in Wilson’s and Menkes diseases [12,17], Alzheimer’s disease [18,19] and cancer PET imaging (e.g. prostate cancer) [20] and radionuclide therapy (e.g. mouse models of malignant melanoma [21]).

One of the main challenges to the wider use of ^64^Cu is the cost and complexity of its production, which is limited to laboratories with cyclotron facilities. ^64^Cu is most often produced via the ^64^Ni(p,n)^64^Cu reaction on a biomedical cyclotron, a route first proposed by Szelecsenyi *et al*. [22] and further developed by McCarthy *et al*. [23]. Due to the high cost of isotopically enriched ^64^Ni (natural abundance 0.926%), alternative ^64^Cu production routes have been explored, including the ^64^Zn(n,p)^64^Cu reaction in a nuclear reactor (which gives low yields and contamination with ^67^Cu) and deuteron irradiation of natural zinc (which produces ^66^Ga and ^67^Ga impurities [24,25,26]).

When using ^64^Cu to radiolabel bioconjugates using bifunctional chelators, high molar activity is often required, and it is important to minimise contamination by nonradioactive trace metals, including nonradioactive carrier copper, which may compete with ^64^Cu for binding to the chelator, interfering with radiolabellng and reducing the molar activity of the radiolabelled product. The ^64^Ni may contain nickel isotopic impurities and other trace metal impurities, including iron and zinc. Metal contaminants can also arise from solutions and equipment used in the production process. Radionuclidic impurities must also be minimised; proton irradiation of ^64^Ni can also produce ^55^Co, ^56^Co, ^57^Co, ^58^Co, ^61^Co, ^57^Ni and ^65^Ni. Purification of ^64^Cu from crude target material is intended to remove such impurities and is typically performed using anion-exchange chromatography using varying concentrations of hydrochloric acid. While many authors have described the production of ^64^Cu [27], few report analytical details of these contaminants, and those reports that do provide such information focus largely on radionuclidic purity (typically by gamma spectroscopy) and molar activity of ^64^Cu [28]. The latter is most often measured by spectrophotometric or HPLC determination using copper chelators such as 1,4,8,11-tetraazacyclotetradecane-1,4,8,11-tetraacetic acid (TETA) [23,28–30] or diacetyl bis(N4-methylthiosemicarbazone (ATSM) [31], or by voltammetry [32], inductively-coupled plasma mass spectrometry (ICP-MS) [28] or ion chromatography [27,28]. Other metal contaminants that may influence labelling and molar activity of the final tracer or indeed interfere with molar activity measurement are typically neglected. More rigorous implementation of processes to minimise and measure this contamination, and quality control criteria, are therefore desirable. Avila-Rodriguez *et al*. [30] were the first to report the analysis by ICP-MS of metal impurities in the final ^64^Cu product. However, access to ICP-MS facilities usually requires that radioactive samples are allowed to decay prior to analysis, incurring delays of weeks.

The objectives of this work were to construct and evaluate a ^64^Cu production system that minimises the amount of costly ^64^Ni for the most cost-efficient production, minimises radionuclidic impurities (particularly ^55^Co) and nonradioactive Cu and other trace metal contamination and maximises radiochemical and radionuclidic purity and molar activity. We report analytical and quality control methods that can be used within typical PET radiochemistry production facilities to measure metal ion concentrations and radiometal molar activities. The methods described have been used to produce ^64^Cu for several published studies [9,12,14,33–45].

## Materials and methods

### General

Reagents and materials were purchased from Sigma-Aldrich (Gillingham, UK) unless otherwise stated. TraceSELECT water for trace analysis was used throughout.

## Methods

### Target backing

**Fig. 1:**
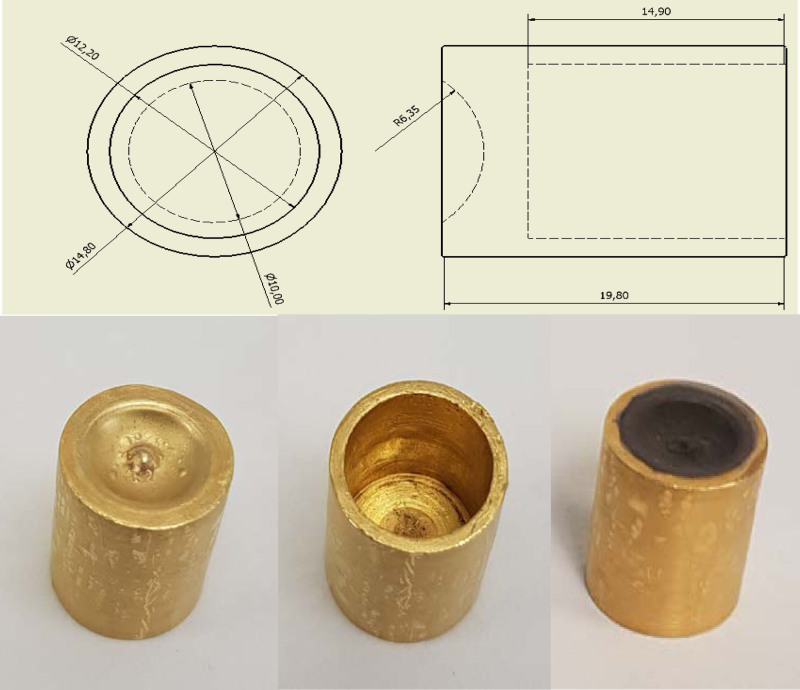
Gold ‘bullet’ target. Top: orthographic projection (dimensions in mm); bottom: view of bullet from front (concave electroplating surface, left) and back (centre), and after electroplating with nickel-64 (right).

The target backing consisted of a 20 mm long ‘bullet’ (Fig. 1) made from high-purity gold (≥99.99%, fine gold grain ARZ000, resistant to the chemicals used to electroplate and dissolve, Cookson Precious Metals, Birmingham, UK) onto which the nickel-64 was electroplated for irradiation. The gold is sufficiently heat-conductive for effective cooling and heating of the surface of the bullet. The surface exposed to the beam was concave with a diameter of 10 mm (equal to the diameter of the incident proton beam) and a radius of curvature 6.35 mm. The bullet is hollow, allowing insertion of a cold (during irradiation) or hot (during dissolution) finger.

### Preparation of the Ni stock solution

^64^Ni (100 mg, 95.6% isotopically enriched, CK isotopes Ltd., Hampshire, UK) was dissolved in a mixture of water (10 mL) and HNO_3_ (10 mL, 70% (15.9 M, ≥99.999% ‘trace metals basis’) in a beaker and heated until evaporated to a green film. The residue was redissolved in HNO_3_ (6 mL, 70% (15.9 M), ≥99.999% trace metals basis) and the evaporation process repeated. After cooling, H_2_SO_4_ (1 mL, 95–98%, 18 M, 99.999%) was added, followed (slowly: CAUTION) by water and the evaporation repeated again. After cooling, the dry residue was dissolved in water, transferred to a volumetric flask and topped up with water to a volume of 50 mL to ensure a final nickel concentration of 2 mg/mL.

### Electroplating

**Fig. 2:**
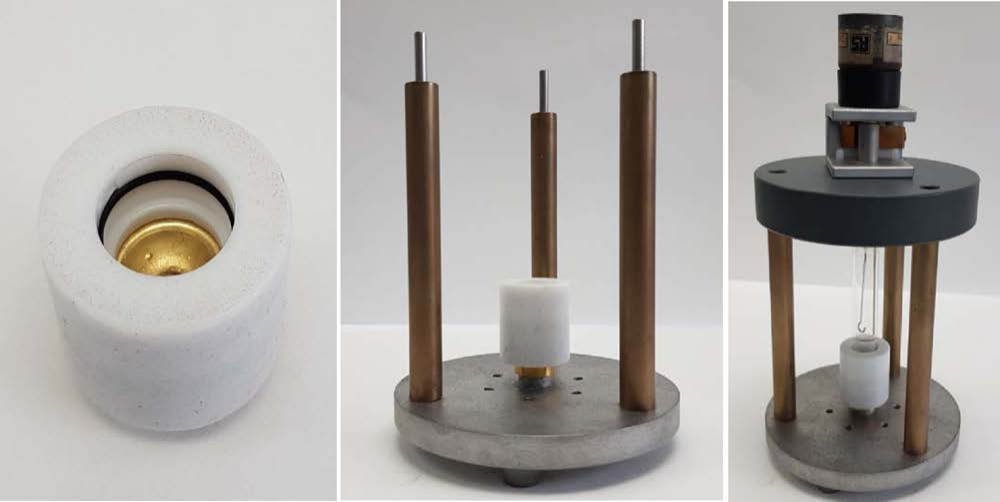
Electroplating cell set-up. Left: gold bullet sealed within PTFE holder; centre: bullet and holder mounted on the electroplating stand; right: with glass cell and platinum anode in place. The gold bullet serves as cathode.

To 5 mL of Ni stock solution prepared as described above (approx. 10 mg of Ni), NH_4_OH (28% NH_3_ in water, 99.9%) was added dropwise until the solution reached pH 9 (approx. 250 µL). (NH_4_)_2_SO_4_ (0.1 g) was then added to this solution before transferring it to the cell for electroplating. The electroplating cell, shown in Fig. 2, consisted of a Pyrex tube (length 7 cm; inner diameter 1 cm; outer diameter 1.2 cm), one end of which was sealed to the preweighed gold bullet (cathode) held inside a poly(tetrafluoroethylene) (PTFE) spacer, on the cathode support plate. The anode was a platinum wire (approx. diameter 1 mm) mounted in the centre of the cell. The cell voltage was slowly increased to a fixed value of 3 V giving initially a 10 mA current and maintained at 3 V for 20 h, during which ^64^Ni was deposited, giving the appearance shown in Fig. 1.

The plated gold bullet was rinsed with water, dried in the oven at 100°C for 30 min, cooled to room temperature and weighed to calculate the amount of Ni deposited. The area of the plated surface was 97 mm^2^, and the calculated thickness of the plated Ni layer was approximately 10 µm.

### Target holder for proton irradiation

**Fig. 3:**
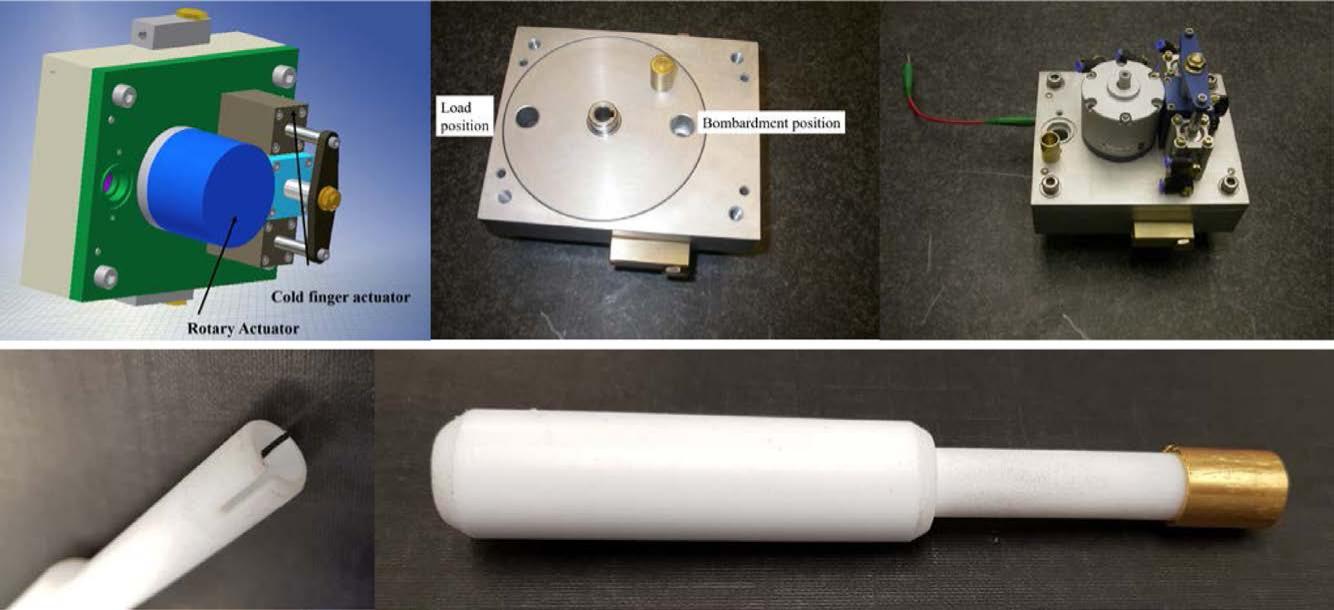
Solid targetry. Top row: Target holder assembly. Left: virtual model; centre: cylinder for holding two diametrically opposed bullets; right: complete assembly. Bottom: hollow PTFE finger used to retrieve bullet from target holder, with (right) and without (left) bullet.

The dimensions of the target holder were designed to be accommodated by the cyclotron shields when closed. The main body carries a rotatable disk with two bores, each of which can accept a gold bullet, set opposite each other along the diameter of the disk (Fig. 3). The bullet can be loaded into the disc at one position, and the disk can be pneumatically rotated by 180° to align the concave surface of the bullet with the cyclotron beam. To the rear of this is a pneumatically-operated cold finger, which, when actuated, makes good thermal contact with the rear inner surface of the bullet for cooling purposes. The cold finger consists of a coaxial cooling pipe with recirculating cooling water from the cyclotron cooling system. Additionally, the surface of the target is cooled by a helium gas flow.

### Ion-exchange column preparation

An empty Bio-Rad column tube (Econo-Column 0.7 × 20 cm) was rinsed with water, followed by 6 M HCl. Biorad AG1-X8 anion-exchange resin (3.5 g, analytical grade, chloride form, 100–200 dry mesh, Bio-Rad, Hertfordshire, UK) was weighed into a beaker and washed with water (2 × 10 mL). The resin was then suspended in 6 M HCl (10 mL), and the resulting slurry was transferred into the column with a glass pipette. The resin was allowed to settle, and excess HCl was drained under gravity.

### Irradiation and target retrieval

The electroplated target prepared as described above was irradiated with protons on a CTI RDS 112 cyclotron (11 MeV, 1 cm beam diameter) with a beam current of 30 μA, for 8 h to induce the ^64^Ni(p,n)^64^Cu reaction. The cold finger was then pneumatically withdrawn, and the disc pneumatically rotated 180 degrees to allow retrieval of the bullet with the aid of a 14 cm long PTFE hollow rod (Fig. 3). The bullet was placed in a lead pot and manually transported to the dose calibrator (CRC-712M, Capintec, Florham Park, New Jersey, USA) to measure the radioactivity after irradiation. The bullet was then placed back in the lead pot and manually transferred to a hot cell equipped with an extractor fan to remove corrosive vapours.

### Target dissolution

**Fig. 4:**
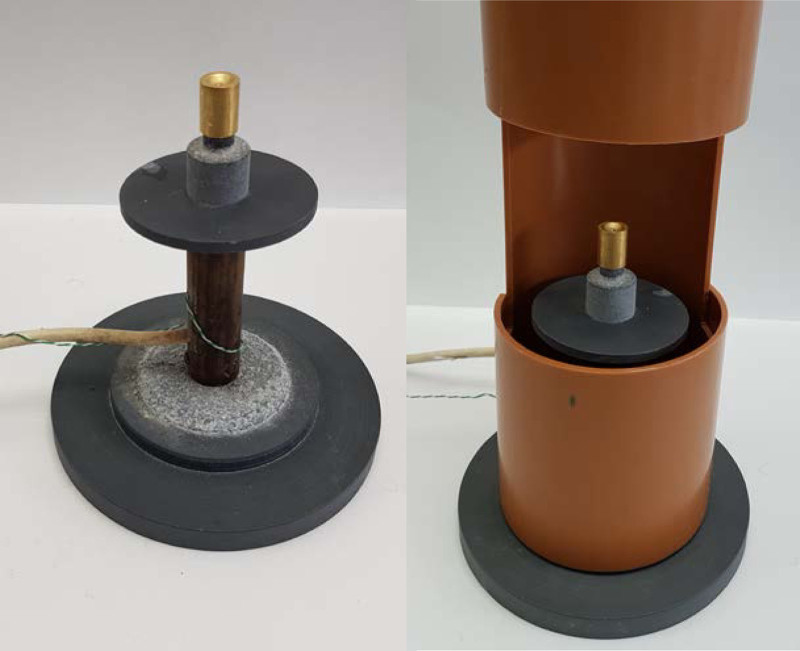
Bullet hot finger assembly with (right) and without (left) extractor hood. The concave surface of the bullet acts as a reaction vessel for the dissolution.

The solid target was placed on the hot finger, which consisted of an aluminium cylinder fitted with a 100 W heater attached to a temperature control box (built in-house, see Fig. 4) set to 90°C (measured using a type K thermocouple). When placed vertically, the concave surface functions as a reaction vessel when dissolving the irradiated nickel, minimising the volume of solvent needed.

9 M HCl (150 μL, prepared from HCl fuming 37% EMPROVE, Merck Millipore, Hertfordshire, UK) was added manually with a glass Pasteur pipette (prewashed with hydrochloric acid and rinsed with water) dropwise to the target at 90°C and allowed to evaporate, increasing the temperature to 95°C if necessary for complete evaporation. After repeating this step 3 times, 9 M HCl (0.3 mL) was added to the target and the dissolved ^64^Ni was manually loaded, using the glass Pasteur pipette, onto an anion-exchange column (AG 1-X8 Anion Exchange Resin, Bio-Rad) prepared as described above. This step was repeated three times, giving a total volume of 0.9 mL added to the column. The column was eluted sequentially under gravity with 6 M HCl (5 mL, prepared by dilution as above) to elute excess ^64^Ni; followed by 4 M HCl (5 mL, prepared by dilution as above) to elute any radiocobalt impurities; and 0.1 M HCl (10 mL, prepared by dilution as above) to elute ^64^Cu. Twenty nominally 1 mL fractions were collected during this process. The final HCl concentration in the eluted fractions does not correspond to the HCl concentration entering the column (i.e. HCl concentration used for elution), as the column has a dead volume of approximately 3 mL.

### Measurement of radioactivity

The total radioactivity of each fraction was measured using a dose calibrator (CRC-712M, Capintec, Florham Park, New Jersey, USA), which had been calibrated for ^64^Cu against the secondary standard ionisation chamber at the National Physical Laboratory (NPL), Teddington, UK.

### Ion chromatography

A method for determination of chloride and trace metals in the fractions by ion chromatography was developed and optimised as described in the Supplementary Information, Supplemental digital content 1, http://links.lww.com/NMC/A191. Fractions with an HCl concentration higher than 0.5 M (fractions 1–15) were diluted to a final concentration of approximately 0.5 M HCl using TraceSELECT Ultra water prior to analysis. Fractions with an HCl concentration equal to or lower than 0.5 M were analysed without any prior dilution. The dilution factors (Supplementary Information Table S1, Supplemental digital content 1, http://links.lww.com/NMC/A191) were calculated by analysing the chloride ion concentration in each fraction with the ion chromatography, using a method for detecting chloride ions. Briefly, this method comprised a Metrosep A Supp 5 column (250/4.0, Metrohm, Runcorn, UK) with 4.8 mM sodium carbonate/1.5 mM sodium hydrogen carbonate as the eluent and 100 mM sulfuric acid as suppressor reagent regenerant. A calibration curve was obtained using samples with chloride ion concentrations in the range of 0.5–10 mM (Supplementary Information Tables S2 and S3 and Figure S1, Supplemental digital content 1, http://links.lww.com/NMC/A191). The dilution factor required to bring chloride concentrations down to within manageable limits for ion chromatography for fractions 1–5 was 12. However, these fractions had to be diluted further in order to bring nickel concentrations down to a level that avoided saturation. Thus, fractions 2 and 3 were diluted not 12× but 1:10 000, fraction 4 1:400 and fraction 5 1:100.

Radiochemical identity and purity of the eluted fractions were determined by injecting 0.1 mL of each of these fractions, after dilution as described in Table S1 and as above, into the ion chromatography system (930 Compact IC Flex, Metrohm) with in-line UV-Vis (944 Professional UV/VIS Detector Vario, Metrohm) and gamma detector (Bioscan Flow-count radio-HPLC) using an Ionpac CS5A column (2 × 250 mm, Thermo Scientific) with 1.4 mM pyridine-2,6-dicarboxylate/13.2 mM potassium hydroxide/1.12 mM potassium sulfate/14.8 mM formic acid (pH 4.0) as the eluent. The flow rate was 0.40 mL/min, and the column temperature was 30°C. The eluate from the column passed through a mixing loop with 1.0 M 2-dimethylaminoethanol/0.50 M ammonium hydroxide/0.30 M sodium bicarbonate containing 0.06 g/L 4-(2-pyridylazo)resorcinol (PAR) at 0.15 mL/min. This postcolumn reagent flow was controlled by the postcolumn delivery system. During this mixing, the metals become complexed with the PAR dye ready for UV-Vis analysis at 530 nm. The delay between the UV-Vis and the gamma detectors was approximately 30 s. Calibration curves (Supplementary Information Tables S4 and S5 and Figure S2, Supplemental digital content 1, http://links.lww.com/NMC/A191) were prepared by analysis of standard solutions ranging from 0.2 to 1.5 mg/L for copper, nickel, zinc and cobalt using TraceCERT atomic absorption standards, 1000 mg/L in nitric acid (Sigma). The method was validated according to the International Conference on Harmonisation guidelines. Calibration data and associated statistics for the determination of trace metals in the fractions are shown in Supplementary Information Table S5, Supplemental digital content 1, http://links.lww.com/NMC/A191).

After analysing all fractions, those that contained most of the pure copper-64 (i.e. fractions 14 and 15), intended for use in radiolabelling, were combined and reanalysed by ion chromatography in order to calculate the molar activity of the [^64^Cu]CuCl_2_. As shown in Supplementary Information Table S1, Supplemental digital content 1, http://links.lww.com/NMC/A191, the HCl concentration of fraction 14 was approximately 2.5 M, while that of fraction 15 was approximately 0.1 M. Combining these two fractions gave an HCl concentration of approximately 1.3 M. An aliquot of this mixture of fractions 14 and 15 was diluted three-fold with TraceSELECT Ultra water prior to analysis to prevent saturation of the ion chromatography detector by HCl.

### Synthesis of [^64^Cu]Cu-ATSM

A C_18_ SepPak Classic cartridge (Waters) was conditioned with absolute ethanol (10 mL), water for injections (10 mL) and air (10 mL), and a 10 mL syringe barrel was attached to the Sep-Pak inlet. ATSM (1 mg, ABX, Radeberg, Germany) was dissolved in anhydrous dimethylsulfoxide (DMSO, >99.9%, 1 mL). This solution (10 µL) was added to the combined fractions 14 and 15 containing [^64^Cu]CuCl_2_ (1703 ± 64 MBq, 1.7 mL) and mixed with the pipette. The reaction mixture was left to stand at room temperature for 15 min and then manually transferred using a glass pipette into the 10 mL syringe barrel attached to the C18 SepPak cartridge. The plunger was inserted into the syringe and used to load the solution onto the cartridge, collecting the eluate in a waste vial. The syringe was detached from the SepPak, and replaced with sequential syringes for washing with water for injections (2 × 20 mL) and air (10 mL). [^64^Cu]Cu-ATSM was then eluted from the cartridge with ethanol in 10 fractions of approx. 0.2 mL each. The fractions containing most of the radioactivity (typically fractions 5–7) were combined, and a 10 mg/mL solution of ascorbic acid in saline was added in sufficient volume (typically approx. 60 µL) to bring the concentration of ascorbic acid to 1 mg/mL. The product was then transferred to an isolator for sterile filtration using a 0.2 µm, 13 mm sterile Millex filter (Millipore) into a nitrogen-filled sterile vial. The minimum volume (typically approx. 5.5 mL) of sterile normal saline was added through the filter to ensure that the ethanol concentration was <10%.

### Half-life measurement

Determination of the radioactive half-life of the final [^64^Cu]Cu-ATSM product was carried out according to the guidelines provided by the British Pharmacopoeia (Radiopharmaceutical preparations, Ph Eur monograph 0125). An aliquot of the product was placed in a dose calibrator (Capintec CRC-712M, calibrated for accuracy for ^64^Cu against the secondary standard ionisation chamber at the NPL, Teddington, UK). The activity thus measured was recorded by a video camera every 30 s over a period of approximately two hours as the product decayed. The natural logarithm of each measured activity was plotted against the measurement time, and a linear model fitted to the data using linear least squares regression. The negative gradient of the regression line is the decay constant (*λ*), from which the half-life can be calculated according to


T12= ln(2)λ (1)

Internal specification for the measured half-life was obtained by applying a tolerance of ±5% to the known ^64^Cu half-life of 12.7 h (i.e. 12.1–13.3 h).

### Radionuclidic purity of [^64^Cu]CuCl_2_ and [^64^Cu]Cu-ATSM

Verification of the radionuclidic purity was carried out using gamma spectrometry [ORTEC GEM Series High-Purity Germanium (HPGe) Coaxial Detector System coupled to a DSPEC jr 2.0 Digital Gamma-Ray Spectrometer]. Calibration of the detector was performed in-house in terms of energy and efficiency by acquiring spectra from a number of sealed and unsealed radioactive sources of known activity positioned in the same calibrated geometry as that used for the radionuclidic purity measurements. The stability of the calibrations was verified regularly using a ^152^Eu source, which has several emission peaks covering the energy range 122–1408 keV. In our standard protocol for radionuclidic purity analysis, spectra of an aliquot of the product were acquired prerelease (immediately after production) and postrelease (approx. 6 h following production) and displayed and analysed with ORTEC GammaVision software (Gamma Vision for Windows Model A66-B32, Version 6.01). During each acquisition, the aliquot was positioned in a calibrated, reproducible geometry at a distance of 50.3 cm from the detector. Background spectra were acquired immediately before each sample acquisition to account for background radiation and gamma shine from other radioactive sources in the laboratory. The prerelease spectrum acquisition time was in most cases limited to approximately 5 min to avoid delaying the release of the product. Postrelease spectra were acquired for approximately 12 h overnight to provide a high sensitivity to low-level impurities. The radionuclidic identity of the sample was verified by observing whether prerelease spectra contained peaks at 511 keV (positron annihilation peak) and 1346 keV (gamma emission with very low abundance of 0.473%, as expected from ^64^Cu).

Radionuclidic purity was calculated as follows: firstly, a standard set of regions of interest (ROIs) was defined covering at least one emission energy from each of the following radionuclides, which are considered to be potential impurities in the ^64^Cu product: ^55^Co, ^56^Co, ^57^Co, ^58^Co, ^60^Co, ^61^Co, ^61^Cu, ^62^Zn, ^65^Zn, ^52^Mn, ^54^Mn, ^65^Ni and ^67^Ni. This ROI set was applied to both the pre- and postrelease spectra and gross and net counts in each ROI extracted using the commercial GammaVision software. These data were then imported into software developed in-house, which calculates the activity of each radionuclide present in the sample (from the background-corrected net counts within each ROI, taking into account the efficiency of the detector at that energy, the yield of the emission and, for the postrelease spectra, the half-life of the radionuclide). Any activities below the critical level as defined by De Geer [46] based on the Currie method [47] were discounted. The radionuclidic purity was calculated from the measured activities of any impurities in the postrelease spectrum and the 511 keV positron annihilation peak in the prerelease spectrum. We applied an in-house specification that the total radioactivity due to radionuclidic impurities must not exceed 0.1%, that is, the radionuclidic purity should be >99.9%, as is standard in the British Pharmacopoeia.

## Methodology used by NPL for radionuclidic purity of [^64^Cu]CuCl_2_

In order to further confirm the radionuclidic identity of the product, an aliquot of [^64^Cu]CuCl_2_ (from the combined fractions 14 and 15 eluted from the ion-exchange column) was analysed by the NPL (Teddington, UK). A 1 g aliquot of the sample was dispensed to a 2 mL ISO ampoule for impurity assessment with a liquid nitrogen-cooled, L-configuration HPGe γ-ray spectrometer manufactured by ORTEC and hereinafter referred to as ‘LISA’. LISA is an n-type HPGe γ-spectrometer housed in a 1.5 × 1 × 1 m lead shield built of 10-cm-thick aged lead with a graded liner comprising of 0.5 mm cadmium and 0.7 mm copper. An aluminium optical breadboard was mounted along the horizontal axis of the detector with a kinematic mounting plate holding a precision-engineered holder allowing highly reproducible source positioning in front of the detector window. The energy calibration of this detector was performed using the most intense γ-rays of a ^152^Eu standard, covering an energy range from 121.8–1408 keV. The spectra were collected using a chain of CANBERRA analogue electronics (AFT Research amplifier 2025, Analogue-to-Digital Converter 8715, aquisition interface module) connected to a PC running the CANBERRA GENIE 2000 v2.1c software. The net peak area losses due to dead time were corrected by using the integrated pile-up rejection (PUR)/live-time correction (LTC) circuit. An additional correction was required for pulse pile-up occurring from random coincidence summing events, which had not been captured by the integrated PUR/LTC circuit of the analogue electronics [48]. This was determined empirically for each detector using the decay-corrected counts of a ^99m^Tc source. All spectra were analysed using CANBERRA GENIE 2000 v2.1c. The fit to each peak was manually reviewed and adjusted where necessary using the CANBERRA GENIE Interactive Peak Fit application. The photopeak areas were corrected for background and decay [49] during the counting period. LISA was calibrated for full-energy peak efficiency using a selection of traceable primary and secondary standards of γ-ray-emitting radionuclides with photon energies between 14 and 1836 keV. The calibration geometry was that of 1 g of aqueous solution in a 2 mL ISO ampoule (same as measurement geometry described above) at a distance of 30 cm from the detector window. Multiple measurements were taken at each calibration point, and at least 10^5^ counts were obtained in each photopeak of interest. All nuclear data used were taken from the evaluations of the Decay Data Evaluation Project [50]. No corrections were made for sample self-absorption or cascade coincidence summing as they were assessed to be insignificant. MDA values were determined using the method described by Currie [46–48].

## Results

Seven batches of [^64^Cu]CuCl_2_ were manufactured and analysed for the purposes of gathering data in this work; many more have been produced for research applications [9,12,14,33–43,45,51]. The average amount of ^64^Ni electroplated for each batch was 9.4 ± 2.1 mg (0.150 ± 0.033 mmol). Irradiation for 8 h produced a measurement of 3.65 ± 0.42 GBq on the dose calibrator with the Cu-64 setting at the end of bombardment (EOB) (Supplementary Information Table S6, Supplemental digital content 1, http://links.lww.com/NMC/A191).

## Ion-exchange chromatography: radioactivity elution profile

**Fig. 5:**
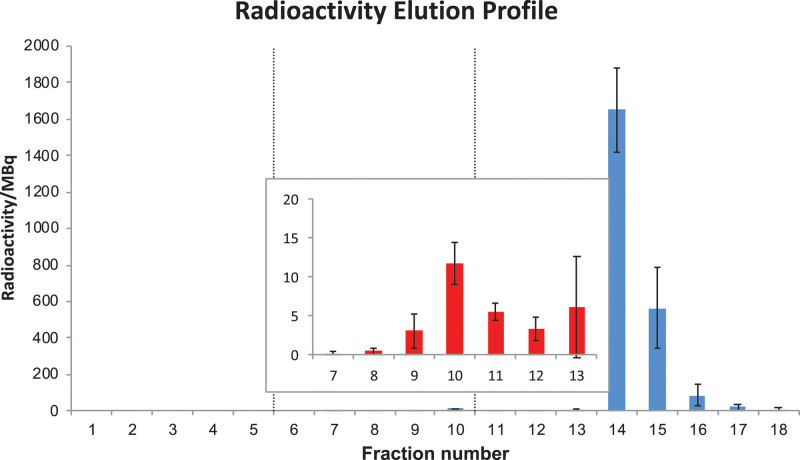
Radioactivity elution profile obtained during the ion-exchange purification of seven representative batches of [^64^Cu]CuCl_2_. Each fraction contains a nominal 1 mL of eluate. Each bar represents the average radioactivity in each fraction collected; error bars represent ±1SD. Inset shows an expansion of fractions 7–13.

**Fig. 6:**
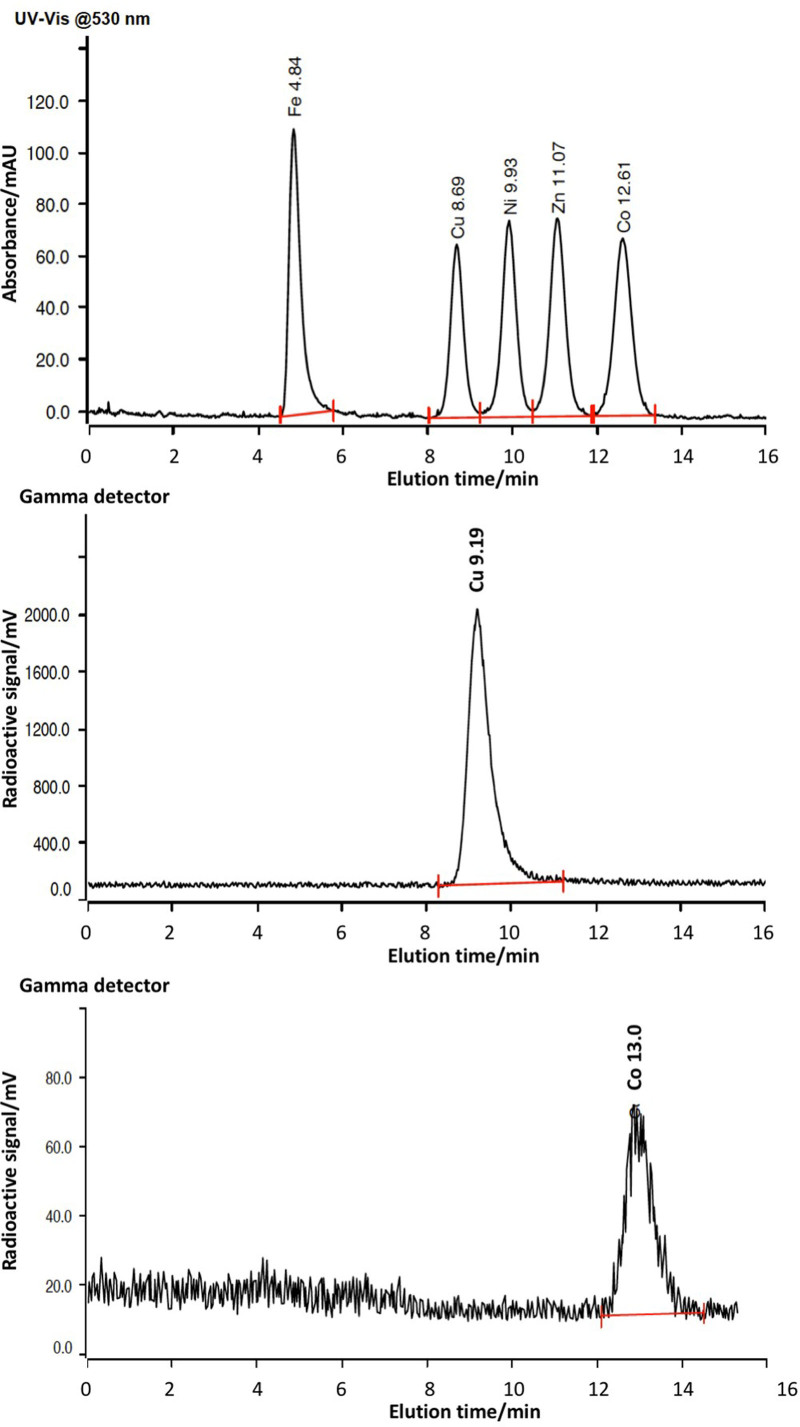
Top: Standard elution ion chromatography profile of Fe, Cu, Ni and Co (visible absorbance 530 nm; concentration 1 µg/mL); centre: typical radio-ion chromatogram obtained from fractions 14–15, showing that all detected radioactivity is attributable to radiocopper; bottom: typical radio-ion chromatogram obtained from fractions 9–11 showing that all detected radioactivity is attributable to radiocobalt. Radiochromatogram elution times are uncorrected for approx. 30 s delay between tandem absorbance and gamma detectors.

Figure 5 shows the average radioactivity in each of the twenty nominally 1 mL fractions collected during the purification of seven different representative batches of [^64^Cu]CuCl_2_, and Fig. 6 shows radio-ion chromatograms of the main radioactive fractions along with calibration standards. The full data are provided in Supplementary Information Table S7, Supplemental digital content 1, http://links.lww.com/NMC/A191).

No radioactivity was detected in the first six fractions. Radioactivity levels increased from fraction 7 to fraction 10 (up to 11.7 ± 2.6 MBq) before decrease in fractions 11–12 (3.4 ± 1.4 MBq, Fig. 5). Radio-ion chromatography of fraction 10 shows that the radioactivity can be accounted for entirely by radiocobalt (^55^Co), eluting at approx. 13 min (Fig. 6). Radioactivity levels increased again in fraction 13 as ^64^Cu began to elute, reaching a maximum in fraction 14 (1650 ± 227 MBq). Most of the ^64^Cu produced was recovered in fractions 14 and 15. These combined fractions contained 2235 ± 221 MBq (i.e. 3159 ± 393 MBq at EOB).

## Ion-exchange chromatography: trace metal elution profile

Details of calibration and sensitivity determination of ion chromatography for a range of trace metal contaminants are provided in Supplementary Information Tables S4 and S5 and Figure S2, Supplemental digital content 1, http://links.lww.com/NMC/A191). The detection limits for Cu, Ni, Zn and Co measured by ion chromatography were 0.04, 0.04, 0.06 and 0.09 µg/mL (0.63, 0.68, 0.92, 1.53 µM) respectively, while the limits for quantification were 0.13, 0.13, 0.18 and 0.27 µg/mL (2.05, 2.21, 2.75, 4.58 µM) respectively.

**Fig. 7:**
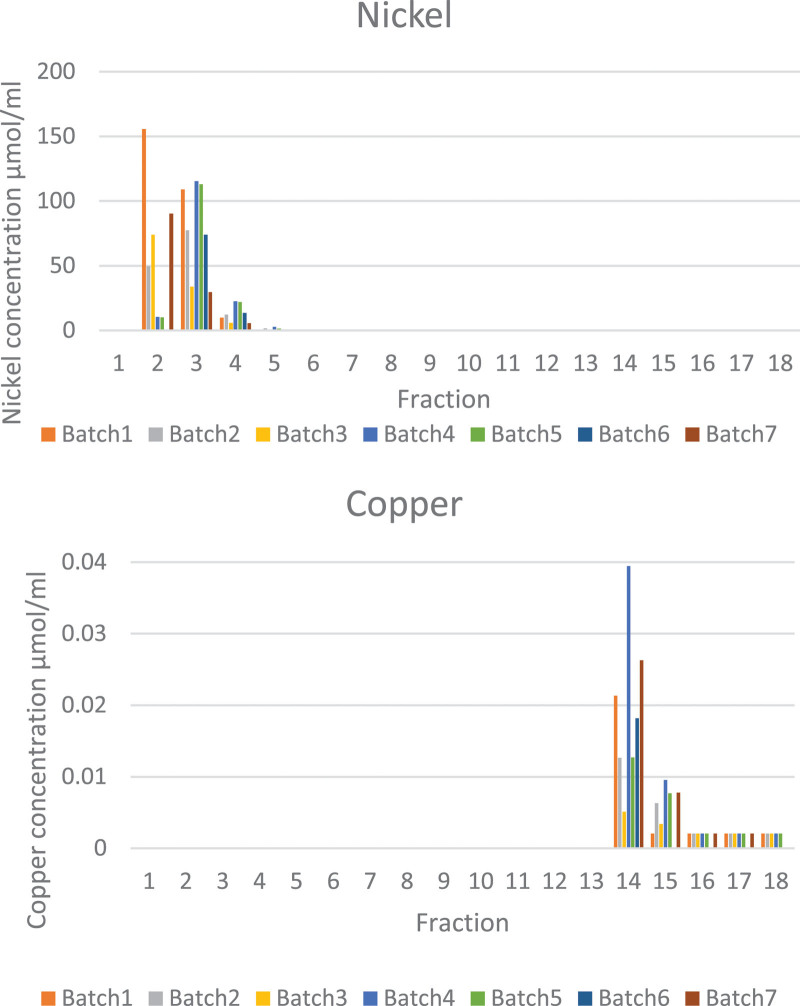
Concentration of nickel (µmol/mL, top) and copper (µmol/mL, bottom) recovered in each of 18 nominally 1 mL fractions during ion-exchange chromatography of 7 representative batches of [^64^Cu]CuCl_2_.

Measured metal concentrations in the fractions eluted from ion-exchange columns during purification of ^64^Cu are shown in Supplementary Information Table S8 and Figure S4, Supplemental digital content 1, http://links.lww.com/NMC/A191). Only nickel and copper were found at concentrations above their detection limits in any fractions from ion-exchange chromatography. Zinc and cobalt were not found above their detection limits in any fractions. Nickel and copper content in all fractions (1–18), as determined by ion chromatography, are shown in Fig. 7. Nickel, originating from the target material, eluted mainly in fractions 2–4, accounting for their light green colour (e.g. chromatogram and data see Supplementary Information Figure S3, Supplemental digital content 1, http://links.lww.com/NMC/A191). The first five fractions, containing on average a total of 0.148 mmol (8.7 mg) of the 0.150 mmol (9.6 mg) nickel originally electroplated onto the bullet (approx. 99%), could therefore be retained for recycling the costly nickel-64. Average nickel concentration in fraction 6 was 100 µM, that in fraction 7 was 20 µM, that in fraction 8 was 13 µM and in later fractions < 8 µM. The very minor (1%) losses in nickel are most likely due to incomplete dissolution of irradiated material from the gold bullet. Copper was found above its detection limit almost exclusively in fractions 14–15 (average copper content in fraction 14 was 19.37 ± 11.18 µM (1.23 ± 0.71 µg/mL); average copper content in fraction 15 was 5.35 ± 3.46 µM (0.34 ± 0.22 µg/mL); average copper content in the combined fraction (14 + 15) was 13.54 ± 6.61 µM (0.86 ± 0.42 µg/mL). Although radio-ion chromatogram analysis of fraction 10 demonstrated that its radioactivity was due entirely to the presence of radioactive cobalt isotopes (^55^Co, eluting at 13 min, see Fig. 6), the UV ion chromatogram of fraction 10 (or indeed any other fraction) showed no detectable cobalt, indicating that the total concentration of cobalt was below the 0.09 μg/mL (1.53 µM) limit of detection.

## Radioanalysis of ^64^Cu-containing fractions 14–15

**Fig. 8:**
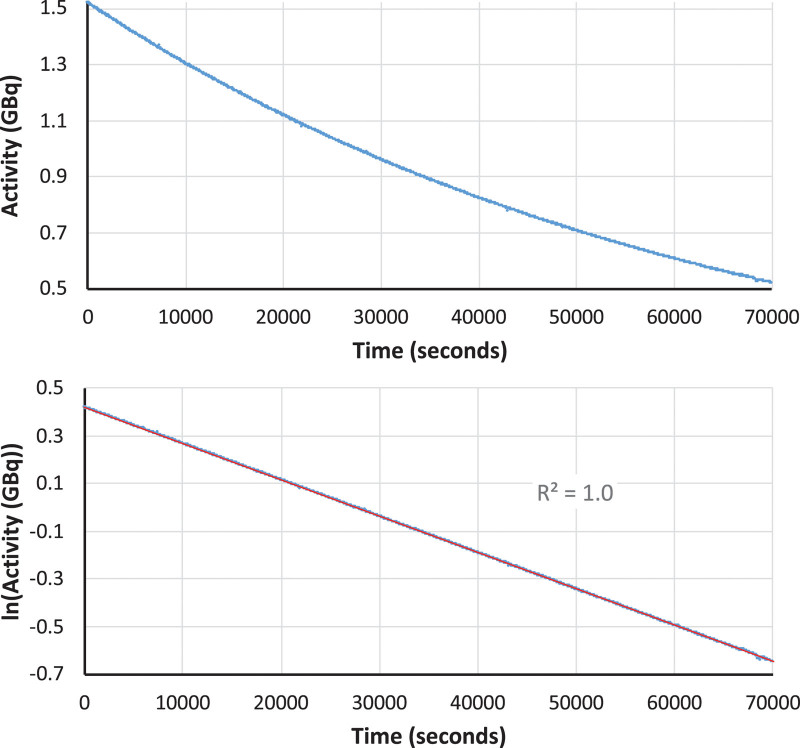
Plot of measured activity against time (top) and plot of the natural logarithm of the measured activity against time (bottom) for a representative fraction 14–15 combination. The line of best fit, shown in red, has a negative gradient (decay constant λ) of 1.525 × 10^−5^ s^−1^.

The combined fractions 14 and 15 were those used for subsequent ^64^Cu radiolabelling work and were therefore subjected to further radiochemical and chemical analysis. A typical radio-ion chromatogram obtained from the analysis of these combined fractions is shown in Fig. 6, along with calibration standards. The presence of a single radioactive peak eluting at ca. 9 min demonstrates that the radioactivity eluted in these fractions was entirely attributable to copper radionuclides. To confirm that this radiocopper was exclusively ^64^Cu, half-life and gamma spectroscopy measurements were performed. Half-life measurements of a representative sample of the combined fractions 14 and 15 are shown in Fig. 8. The half-life calculated from these data was 12.6 h. This is well within the specified limits, identifying the radionuclide as ^64^Cu without measurable radionuclidic impurity.

**Fig. 9:**
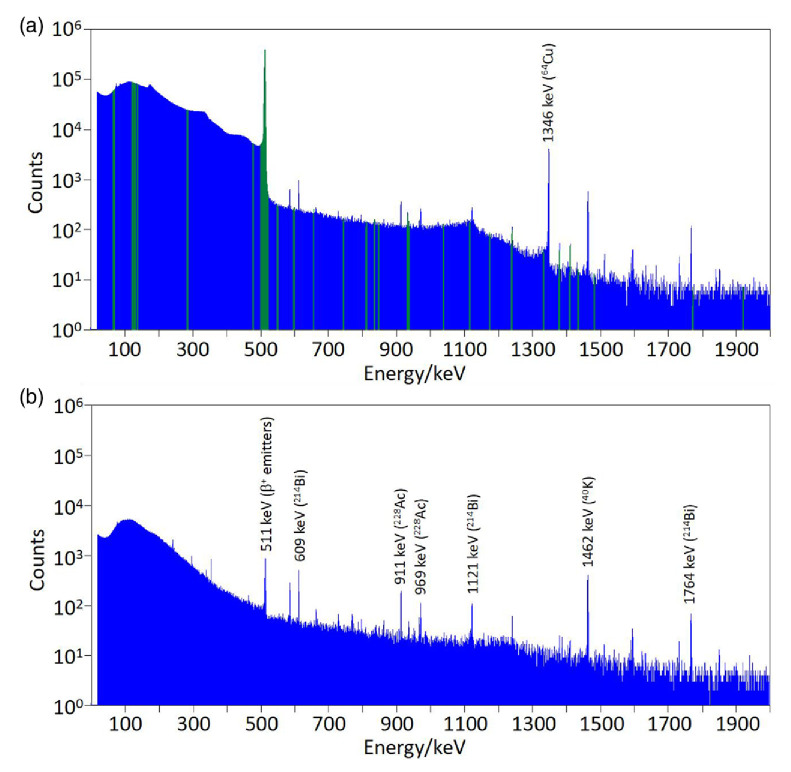
(a) Gamma spectrum from an exemplar [^64^Cu]CuCl_2_ batch (acquisition time 43 200 s). The 1345.8 keV emission from ^64^Cu is present among the background peaks. The green sections indicate the ROIs used to identify impurities for the radionuclidic purity calculation. (b) Background spectrum obtained overnight with no sample present (acquisition time 28 800 s). ROIs, regions of interest.

Radionuclidic purity and identity measurements were performed using gamma spectroscopy on both [^64^Cu]CuCl_2_ samples directly eluted in fractions 14–15, and [^64^Cu]Cu-ATSM subsequently produced from these fractions. An example of a spectrum from [^64^Cu]CuCl_2_ is shown in Fig. 9, together with a background spectrum. The ROIs shown in green are those used to calculate the radionuclidic purity. The 1346 keV peak present in the sample spectrum, characteristic of ^64^Cu, is absent from the background spectrum. This peak is a unique identifier of ^64^Cu, avoiding uncertainty due to the potential presence of other positron-emitting radionuclides in the samples and the environment (511 keV in our HPGe spectra from other positron-emitting radionuclides contributing to the background radiation flux in the laboratory could not be completely eliminated). Radionuclidic purity was calculated from these spectra for seven batches of [^64^Cu]CuCl_2_. Results are shown in Table 1. Background correction was carried out using background spectra acquired on the day of production; or alternatively using the overnight background spectrum shown in Fig. 9. The acquisition time of the sample spectra used for the radionuclidic purity calculation varied from 1 to 12 h, as indicated in Table 1. All batches had a radionuclidic purity >99.95% at the time of analysis. Correction of the calculated radionuclidic purities to the end of beam time did not significantly affect the results, and all values remained above 99.9%.

**Table 1:** Radionuclidic purity results for seven batches of [^64^Cu]CuCl_2_

Batch number	Sample acquisition time (s)	Radionuclidic purity at time of analysis (%)
160608-03 (NPL sample)	3600	99.999
160511-03	5813	99.987
160406-03	43 200	99.993
160413-04	43 200	99.975
160420-05	43 200	99.984
160824-06	36 000	99.976
161102-05	36 000	99.976

The radionuclidic purity was typically measured between 7 and 10 h after end-of-bombardment (EOB). Batch numbers are recorded for internal reference.

One [^64^Cu]CuCl_2_ sample (batch 160608) was analysed by the NPL, identifying the presence of a single radioactive contaminant, ^55^Co, with an activity of 33.5 ± 7.9 kBq at midday on the day of production. This corresponds to a radionuclidic purity of 99.998% at midday on the day of production (i.e. 7 h after EOB). The calculated radionuclidic purity does not change when decay-corrected to the end of the bombardment. This is above the 99.9% specification.

Gamma spectroscopy measurements were also carried out on samples of [^64^Cu]Cu-ATSM prepared from the [^64^Cu]CuCl_2_. No peaks characteristic of ^55^Co or other peaks not attributable to ^64^Cu were detected in background-corrected spectra.

## Chemical analysis of ^64^Cu-containing fractions 14–15

The total concentration of copper, determined by ion chromatography, in fraction 14 ranged from 5 to 39 µM, and in fraction 15 from below the quantification limit 2.05–9.55 µM (after correcting for dilution). Fractions 14 and 15 were combined, diluted three-fold with TraceSELECT Ultra water and reanalysed. The average concentration of copper in fractions 14–15 combined, after correcting for the dilution, was 13 ± 6 µM, yielding a molar activity of 121.5 ± 32.7 GBq/µmol corrected to EOB. The average concentration of nickel, determined by ion chromatography, in fractions 14 was 11.9 µM (0.76 µg/mL), while that in fractions 15 was 2.0 µM (0.13 µg/mL). The concentration of zinc in fractions 14 and 15 was below the quantification level (2.7 µM, 0.18 µg/mL) while no cobalt was detected by ion chromatography (detection limit 1.53 µM (0.09 µg/mL)). In the combined ^64^Cu fractions 14 and 15 used for radiolabelling, ion chromatography demonstrated the presence of nonquantifiable traces of iron.

Ion chromatography analysis of some of the other collected fractions revealed the presence of small amounts of zinc and iron contaminants that are often present in the environment. The amount of zinc found in each fraction varied from batch to batch without following a consistent pattern, suggesting that its presence was due to environmental contamination (see discussion section). Zinc is a ubiquitous contaminant, and the fact that glassware used in this experiment was not washed with acid could have most likely contributed to the presence of this metal in the fractions.

Subsequent uses of the ^64^Cu produced may be affected by the chloride concentration. Although fraction 14, containing the majority of the ^64^Cu, was eluted after the eluent had been changed to 0.1 M HCl, its chloride concentration (measured by ion chromatography) was typically 2.5 M; by fraction 15, the equilibrium concentration of 0.1 M was reached as shown in the Supplementary Information Table S1, Supplemental digital content 1, http://links.lww.com/NMC/A191). Consequently, the average chloride concentration in the combined fractions 14–15 was 1.3 M.

## Discussion

The aims of this work were to develop a method for the production of ^64^Cu for a variety of applications, to develop a methodology for validating its quality (yield, radionuclidic purity, specific/molar activity, trace metal contamination, etc.) and report the values of these standard quality parameters. Among methodology and analysis so far reported for ^64^Cu, including commercially available material with marketing authorisation from the regulatory agencies, this work is the most comprehensive. Previous publications describing the use of ^64^Cu rarely give specifications of purity, molar activity, etc.

The gold bullet used as the target backing was custom-made in-house using high-purity (≥ 99.99%) gold. The use of gold as a backing material entails high capital cost but offers advantages such as low activation and the possibility of reusing the same target backing for many years. The amount of nickel required for electroplating to ensure the formation of a homogeneous layer of ^64^Ni depends on the shape and dimensions of the target backing. The dimensions of the gold bullet used in this study allowed us to minimise the amount of costly ^64^Ni required for successful production and to minimise the volume of solution added to the ion-exchange column, and hence the volume of the ^64^Cu-containing fractions eluted from it. The latter is important for preclinical work because of the limited volumes that can be injected into mice. The area of the plated surface of the gold bullet was 97 mm^2^, and the amount of ^64^Ni plated for each production was approx. 10 mg, which results in an average thickness of the plated ^64^Ni layer of approx. 10 μm. By contrast, most authors report plating larger amounts of ^64^Ni ranging from 60 to 100 mg (Supplementary Information Table S11, Supplement digital content 1, http://links.lww.com/NMC/A191) [27,29]. Electroplating was performed using a platinum wire as the anode. In the early development of this methodology, a motor to rotate the platinum wire was used during electrodeposition to agitate the solution and maintain a flow of fresh electrolyte to the gold bullet surface. However, we found no difference in electrodeposition efficiency or the regularity of the layer when rotation was omitted. Rotation is therefore no longer used.

After irradiation, the gold bullet is extracted from the cyclotron, and ^64^Cu and the electroplated ^64^Ni, along with other impurities, are recovered from the bullet with 9 M HCl. As mentioned earlier, the concave surface of the bullet acts as a reaction vessel, minimising the total volume of HCl required for dissolving the irradiated material to <1 mL. Other target backing designs [23] require larger volumes of acid as they need to be immersed in a dissolution vessel containing HCl for the irradiated material to be dissolved. Moreover, when using a conventional dissolution vessel in which the entire target is immersed, care must be taken to clean the back of the target prior to sample dissolution to avoid poor molar activity due to contamination from the target cooling water, as reported by McCarthy *et al*. [23]. The concave ‘bullet’ system avoids this difficult manipulation. The small volume is highly advantageous: it saves the inconvenient evaporation step and facilitates easier buffering for subsequent radiolabelling. In order to avoid introducing contaminants, high-purity grade hydrochloric acid and metal-free water are essential. Similarly, the use of metal tools must be avoided, and attention must be paid to the material and cleanness of containers used to transport the irradiated bullet from the cyclotron to the production hotcell.

The enriched ^64^Ni target material is a potential source of trace metal impurities. The isotopic and chemical purity of commercially available ^64^Ni varies between batches, which may contain several nickel isotopes. The isotopic composition of the ^64^Ni used in work presented here is summarised in the Supplementary Information Table S12, Supplemental digital content 1, http://links.lww.com/NMC/A191). Depending on the relative ratio of the nickel isotopes present in the target material, nickel and cobalt radioisotopes such ^57^Ni, ^55^Co, ^56^Co, ^57^Co, ^58^Co, ^61^Co, along with ^64^Cu, can be produced during irradiation (Supplementary Information Table S13, Supplemental digital content 1, http://links.lww.com/NMC/A191). Since nickel and cobalt impurities were shown to be efficiently removed by the ion-exchange purification process, these potential contaminants do not represent a significant problem either for experimental or clinical use of the product or for radioactive waste disposal. Other chemical impurities present in the ^64^Ni target include metals such as aluminium (typically 20 ppm), cadmium (<10 ppm), cobalt (<60 ppm), chromium (30 ppm), iron (50 ppm), lead (10 ppm) and zinc (<50 ppm) could, in theory, give rise to contaminating radionuclides (Supplementary Information Table S14, Supplemental digital content 1, http://links.lww.com/NMC/A191), but none were detected.

The separation mechanism during ion-exchange chromatography is based on the varying extent of formation of anionic chloro-complexes of the metals in this hydrochloric acid medium, according to the Eqs. 2–5 below (which ignore the presence of coordinated water molecules):


$${\text{M}}^{2+}+{\text{Cl}}^{-}⇌{\text{MCl}}^{+}$$ (2)

$${\text{MCl}}^{+}+{\text{Cl}}^{-}⇌{\text{MCl}}_{2}$$ (3)

$${\text{MCl}}_{2}+{\text{Cl}}^{-}⇌{\text{MCl}}_{3}^{-}$$ (4)

$${\text{MCl}}_{3}^{-}+{\text{Cl}}^{-}⇌{\text{MCl}}_{4}^{2-}$$ (5)

where M^2+^ is the divalent transition metal ion. The formation of cationic, neutral or anionic species depends on the formation constant for each individual step, the preferred coordination number of the metal and the concentration of chloride ions. In the 9 M HCl used here, MCl_3_^−^ and MCl_4_^2−^ complexes predominate and will bind to the anion-exchange column, allowing ^64^Cu to be purified from ^64^Ni and other cold and/or radioactive metal contaminants by anion-exchange chromatography using AG 1-X8 resin, a strongly basic anion exchanger with quaternary ammonium functional groups attached to the styrene divinylbenzene copolymer lattice (R-CH_2_N^+^(CH_3_)_3_), supplied in the chloride form. The anionic metal chloro-complexes are retained by the resin according to the equilibria shown below:


$$\begin{array}{l} \text{R}-{\text{CH}}_{\text{2}}{\text{N}}^{+}{\left({\text{CH}}_{\text{3}}\right)}_{\text{3}}-\;Cl^{-}\;\;+\;\mathrm{MC}I_{3}^{-}\;\;⇌\\ \text{R}-{\text{CH}}_{\text{2}}{\text{N}}^{+}{\left({\text{CH}}_{\text{3}}\right)}_{\text{3}}-\;\mathrm{MC}I_{3}^{-}\;\;+\;Cl^{-} \end{array}$$ (6)

$$\begin{array}{l} \text{2(R}-{\text{CH}}_{\text{2}}{\text{N}}^{+}{\left({\text{CH}}_{\text{3}}\right)}_{\text{3}}-\;Cl^{-}\;\;+\;\mathrm{MC}I_{4}^{2-}\;\;⇌\\ \text{R}-{\text{CH}}_{\text{2}}{\text{N}}^{+}{\left({\text{CH}}_{\text{3}}\right)}_{\text{3}}-\;\mathrm{MC}I_{2}^{4-}\;\;+\;2Cl^{-} \end{array}$$ (7)

MCl_2_ species and MCl^+^ cations do not interact with the resin. Separation is achieved by eluting the metal chloro-complexes with HCl at decreasing molar concentration. As the HCl concentration decreases, the equilibria shown above (2–5) shift to the left, changing the net charges of the various metal chloro-complexes from negative to neutral or positive, hence releasing the complexes from the resin sequentially, in agreement with the results from Kraus *et al*. [52] (Supplementary Information Figure S4, Supplemental digital content 1, http://links.lww.com/NMC/A191). Sequential elution of the column with 6 M, 4 M and 0.1 M HCl used in our experiments achieved the separation of nickel, cobalt and copper, respectively, without significant overlap.

The optimisation of the ion-exchange purification is described in Supplementary Information, Supplemental digital content 1, http://links.lww.com/NMC/A191. Nickel was the first metal to elute from the column. It has been reported that nickel does not interact with anion exchangers because negatively charged complexes are not formed in appreciable amounts, even in highly concentrated hydrochloric acid [53]. In hydrochloric acid solutions up to 3 M, most of the nickel exists as hydrated nickel ion [54], with the mole fraction of Ni^2+^ ion decreasing with increasing HCl concentration, to favour an increase in the mole fraction of NiCl^+^ but not NiCl_2_, NiCl_3_^−^ or NiCl_4_^2−^. Hence, we were able to collect most of the ^64^Ni in the first 4 fractions by using 6 M HCl. The chromatograms acquired from these fractions by radio-ion chromatography did not show any radioactive signal corresponding to the nickel or copper peaks; therefore, no ^57^Ni was produced during irradiation, and ^64^Cu was fully retained on the column at this stage in the elution. Other metals sometimes present in these fractions were iron and zinc (although accurate quantification of these was not possible as the concentration in the analysed diluted fractions was below the detection and/or quantification limit), while cobalt and copper were not detected by ion chromatography. Iron and zinc chloro-complexes are strongly retained by the resin under these elution conditions. In fact, even at a concentration of 3 M HCl, more than 90% of the zinc is present in the form of ZnCl_4_^2−^ and ZnCl_3_^−^ [55], and is expected to remain adsorbed even at 0.1 M HCl. Thus, the presence of zinc in these early fractions eluted with 6 M HCl is most likely due to postcolumn environmental contamination, for example, from the glass vials used for fraction collection and glass pipettes used for the addition of the mobile phase to the column. As many authors have previously indicated, this highlights the need for washing all the glassware in acid and rinsing it with metal-free water prior to use. This is not only important to avoid metal contamination in fractions containing ^64^Cu, but also for earlier fractions to allow the optimum recycling of uncontaminated ^64^Ni collected in the first fractions.

The removal of ^64^Ni with highly concentrated hydrochloric acid has been described by many authors [24,26,27,29–31]. However, upon complete elution of nickel, many reported processes for ^64^Cu purification incorporate comparatively low concentration of HCl (0.5 or 0.1 M [23,27,29]) to elute the ^64^Cu at this stage, resulting in the poor separation of radioactive cobalt isotopes from ^64^Cu. To reduce contamination by radiocobalt, some authors discard the early part of this volume [27] (where cobalt should be mostly contained) which risks discarding part of the ^64^Cu produced. Our procedure incorporates an additional intermediate (4 M) hydrochloric acid concentration to elute cobalt, which at this concentration is mainly in neutral or cationic form while most of the copper is still anionic and bound to the resin. Upon complete elution of cobalt, the elution of copper was accelerated by switching the mobile phase to 0.1 M, rendering the Cu cationic and eluting it free of radiocobalt.

The majority of the activity produced after irradiation was consistently recovered in fractions 14 and 15, in which ^64^Cu was the only radionuclide detected by radio-ion chromatography. Gamma spectrometry showed that other radiometals and other radioisotopes of copper were absent. Thus, these fractions had high radiochemical and radionuclidic purity and were suitable for radiolabelling applications. The main nonradioactive metallic contaminant was copper, reducing molar activity to the extent that ^64^Cu represented roughly 1 in 75 copper atoms at the end of the bombardment, comparable with other reported methods (Supplementary Information Table S15, Supplemental digital content 1, http://links.lww.com/NMC/A191). Other contaminating divalent metal cations, which can affect radiolabelling by competing to bind to bifunctional chelators, were as low as reasonably achievable.

Radiolabelling of the ATSM ligand with the ^64^Cu produced was performed as previously described. The crude [^64^Cu]Cu-ATSM was purified using a C18 cartridge, minimising unreacted [^64^Cu]CuCl_2_ and nonradioactive contaminants in the final product. The ^64^Cu produced in this way has also been used efficiently in biomolecule labelling applications using bifunctional chelators, where high purity and molar activity are more critical to success.

Automation of the procedures as far as possible would be preferable from the point of view of operator dosimetry, but this was not possible during the development of the method presented here. The work was carefully risk-assessed from the point of view of operator radiation doses, and all operators were monitored during every procedure to ensure their doses remained within constraints. Shielding was used to good effect to minimise doses as far as possible. The finger, whole body and eye doses recorded for everyone involved in the project were comfortably within regulatory limits. This was successfully achieved by limiting either the frequency of productions or the frequency at which a given operator performed the procedures. This was possible due to the lack of requirement for routine production of [^64^Cu]CuCl_2_ or [^64^Cu]Cu-ATSM, which made it viable to adjust the time between consecutive productions. However, if routine production were required, especially for clinical use where higher activities are required, further development of the process should and would consider the automation of certain steps in order to reduce operator dosimetry.

### Conclusion

We have described optimised methods for the production of ^64^Cu in batches of ca. 3.2 GBq, in a volume of solution (<2 mL) that is sufficiently small to be readily amenable to preclinical imaging studies in mice, with stringent specifications for specific/molar activity (ca. 121.5 ± 32.7 GBq/µmol) and trace metal contamination. We have described analytical methods for validation and quality control that can be used within the radiochemistry facility. The methods and data serve as standards against which future ^64^Cu production, and use of ^64^Cu for radiochemical, preclinical and clinical research, can be referenced.

## Acknowledgements

This work was supported by the Centre of Excellence in Medical Engineering funded by the Wellcome Trust and the Engineering and Physical Sciences Research Council (EPSRC) under grant number WT088641/Z/09/Z, the King’s College London and University College London Comprehensive Cancer Imaging Centre funded by Cancer Research UK and EPSRC in association with the Medical Research Council and Department of Health (England), the National Institute for Health Research Biomedical Research Centre based at Guy’s and St. Thomas’ NHS Foundation Trust and King’s College London (KCL) and a grant (S60389) from the EPSRC.

## Conflicts of interest

There are no conflicts of interest.

## Supplementary Material


