# Supplementary material for: Production of copper-64 using a hospital cyclotron: targetry, purification and quality analysis
Source: Nucl Med Commun. 2021 May 5;42(9):1024–38. doi: 10.1097/MNM.0000000000001422 (PMC8357037; doi:10.1097/MNM.0000000000001422)

Figure 1s

A 48-year-old patient with lung cancer, example of standardized uptake value (SUVpeak, defined as the largest possible mean value of a 1cm3 regions of interest) and manually drawn regions of interest of global lung glycolysis measurement and total lesion glycolysis in axial, sagittal, coronal PET/CT fusion images(A, B, and C, respectively).The yellow outlined the affected side, white color means tumor region

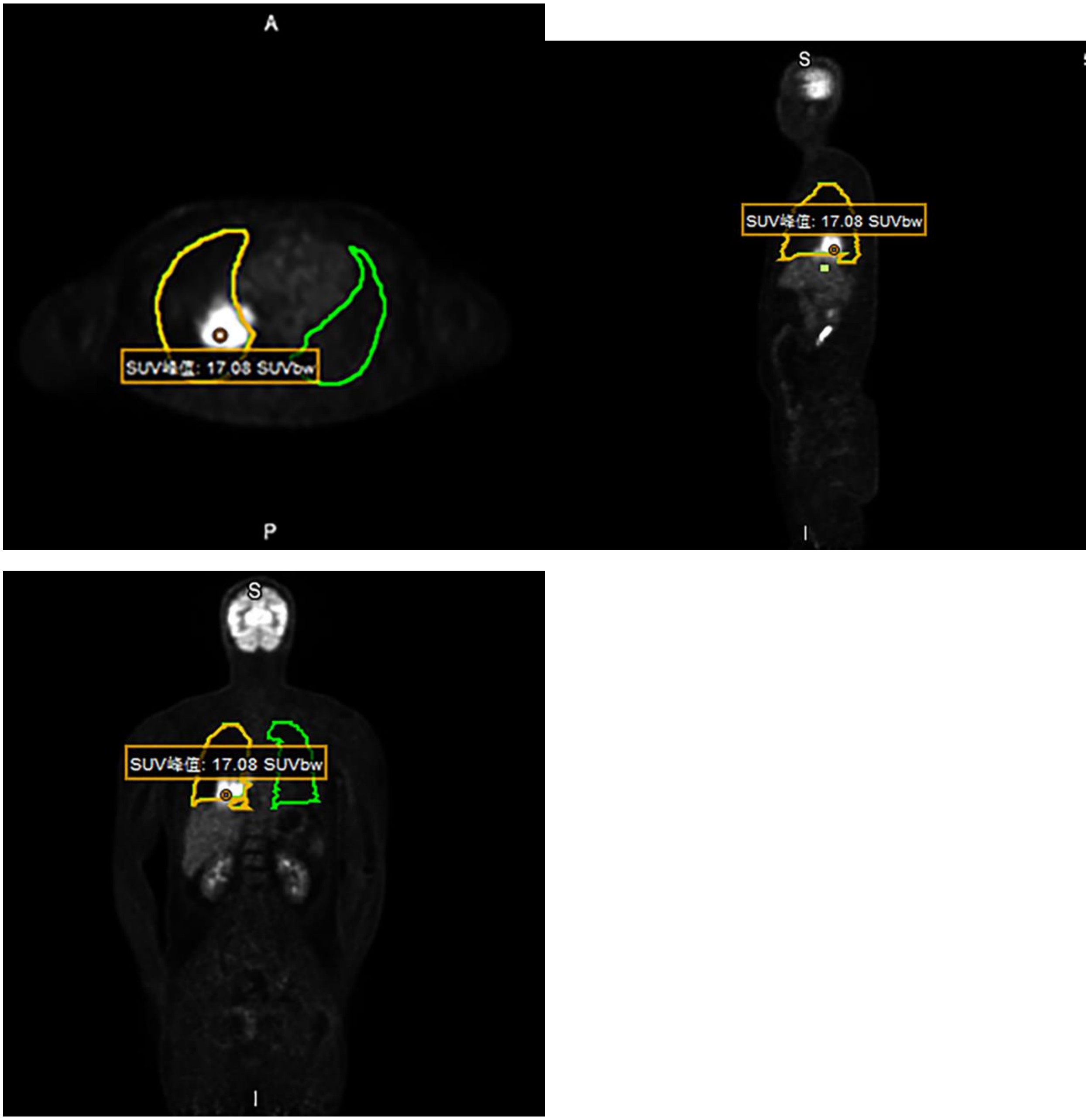

Supplement: Supplementary file 1 [file nmc-42-1024-s001.pdf]
